# Predicting the characteristics of a C_2_B_6_ monolayer with ultrahigh carrier mobility

**DOI:** 10.3389/fchem.2024.1482006

**Published:** 2024-10-24

**Authors:** Ping Xu, Zhengyang Zhu, Ruxin Zheng, Qingyun Sun, Zhen Ma, Weihua Mu, Zhen Cui

**Affiliations:** ^1^ Jiangsu Vocational College of Agriculture and Forestry, Jiangsu, China; ^2^ School of Mechanical Engineering, Wanjiang University of Technology, Maanshan, China; ^3^ School of Mechanical Engineering, Southeast University, Nanjing, China; ^4^ School of Mechanical and Electronic Engineering, Nanjing Forestry University, Nanjing, Jiangsu, China; ^5^ Nanjing Boya Intelligent Technology Co., Ltd, Jiangsu, China; ^6^ School of Agricultural Engineering, Jiangsu University, Zhenjiang, China; ^7^ Wenzhou Institute, University of Chinese Academy of Sciences, Wenzhou, China; ^8^ School of Automation and Information Engineering, Xi’an University of Technology, Xi’an, China

**Keywords:** two-dimensional, first principle calculations, C2B6, mobility, optoelectronics

## Abstract

Two-dimensional materials have excellent electronic and optical properties, suggesting absolute advantages in nanodevices. In this work, a new two-dimensional material with a puckered structure, a C_2_B_6_ monolayer, is proposed. The material presents dynamic and thermal stability calculated by first-principle simulations. Interestingly, the C_2_B_6_ monolayer possesses semiconductor behavior with an ultra-narrow bandgap of approximately 0.671 eV by HSE06 functional. Meanwhile, the hole in the C_2_B_6_ monolayer shows ultrahigh mobility at approximately 6,342 cm^2^⋅V^−1^⋅s^−1^ in decent transport directions, which is larger than traditional transition metal dichalcogenides materials. More importantly, the pronounced anisotropy of mobility of the electrons and holes can separate the photogenerated charges, suggesting the applications for photocatalytic, photovoltaic and optical and cold chain electronic devices. Then, the novel properties of the light absorption characteristic are obtained, and the anisotropic photocurrent implies the C_2_B_6_ monolayer can be used as a potential photoelectric device. Our results provide theoretical guidance for the design and application of two-dimensional materials.

## Introduction

Since the discovery of graphene (Geim and Novoselov, 2007), there has been an increasing amount of research on two-dimensional (2D) materials (Miro et al., 2014; Ren et al., 2024). The wide application of the unique properties and advantages of 2D materials has made them highly regarded research in the field of materials science (Tang et al., 2022; Wang et al., 2023; Su et al., 2022; Su et al., 2023; Sun et al., 2022). For example, due to the extremely thin thickness of transition metal dichalcogenides (TMDs), their light absorption performance is outstanding, suggesting potential applications in fields such as solar cells and optoelectronic devices (Zhao et al., 2024; Ren et al., 2019). The AlN monolayer also has outstanding strength and stiffness in the plane direction compared with the bulk one (Ren et al., 2021a; Ren et al., 2021b). In addition, the larger specific surface area exposes more catalytic active sites; therefore, 2D materials present excellent photocatalytic and electrocatalytic properties.

Wu et al. (2020) prepared IrPdPtRhRu high-entropy alloy (HEA) nanoparticles with a mean diameter of 5.5 ± 1.2 nm by a facile one-pot polyol method, which possesses a lattice constant of 3.856 Å. The HAADF-STEM configurations of the IrPdPtRhRu HEAs and the corresponding energy-dispersive X-ray (EDX) images of each element suggest the solid-solution alloys obtained by homogeneous distribution. The duration of the IrPdPtRhRu was evaluated in both acidic (0.05 M H_2_SO_4_) and alkaline (1.0 M KOH) electrolytes, which proves the hydrogen evolution reaction (HER) ability of the IrPdPtRhRu HEA NPs.

High throughput computing method investigations are conducted to develop new 2D materials, expand their application, and develop more novel mechanical, optical, and electronic properties (Ren et al., 2022a; Sun and Schwingenschlögl, 2021; Haastrup et al., 2018). For example, Luo used particle swarm optimization to structure boron carbon compounds, and the results show that boron carbon compounds have strong B–C bonds and thermal stability and can maintain structural stability even above 2,000 K (Luo et al., 2011). Lu proposed a CaP_3_ monolayer with a direct bandgap of approximately 1.15 eV, and the electron mobility obtained is as high as 19,930 cm^2^⋅V^−1^⋅s^−1^ (Lu et al., 2018). Yuan predicted the monolayered penta-RuS_4_ through first-principle calculations, and interestingly, this monolayered penta-RuS_4_ structure exhibits unique anisotropic secondary energy dispersion (Yuan et al., 2017). Jing presented a monolayered GeP_3_ crystal that has an indirect bandgap of 0.55 eV. The double-layer GeP_3_ possesses a decreased bandgap of 0.43 eV. It is noteworthy that the GeP_3_ monolayer can transform the indirect bandgap into the direct bandgap under the condition of biaxial strain. Meanwhile, GeP_3_ also has remarkable light absorption ability and can be widely used in optoelectronics (Jing et al., 2017).

Jin proposed a novel Janus MoTe monolayer using density functional theory (DFT). The results indicate that the monolayered Janus MoTe presents relatively wide spatial extension and low binding energy. Furthermore, the time for electron–hole recombination is approximately 1.31 ns, making it a potential photocatalyst for water splitting (Jin et al., 2018). More recently, researchers used the B_2_P_6_ present Janus structure and proved that B_2_P_6_ is an indirect bandgap semiconductor with an excellent hydrogen production efficiency of 28.2% and an outstanding photocatalyst (Sun and Schwingenschlögl, 2020a) that also can be tuned by external strain (Ren et al., 2021c). For a B_2_P_6_ monolayer, the HER and oxygen evolution reactions (OERs) can be induced respectively at different surfaces because the energy levels of the two surfaces exhibit staggered band energy, thereby separating the photogenerated electrons and holes. Such a Janus structure of the B_2_P_6_ monolayer exhibits intrinsic differences by atomic adsorption on different surfaces (Ren et al., 2022b).

A CS monolayer was proposed with strong absorption of solar radiation and conversion efficiencies as high as 20.1% (Sun and Schwingenschlögl, 2020b), which also presents decent band edge positions for the redox reaction in water splitting used as a photocatalyst. A CN monolayer shows a wide bandgap of approximately 6 eV as a potential power device (Ren et al., 2023a). The wide bandgap and extremely strong elastic modulus of the CN monolayer enable it to maintain the potential for photocatalytic water splitting even under large strains. Thus, B- or C-atom-based new materials are proposed to possess novel electronic and optical performances for use in nanodevices.

In this investigation, a novel monolayered C_2_B_6_ system is proposed by the elemental mutation method considering the prototype of the Li_*x*_B_*y*_ structure. Using the first-principle calculations, the C_2_B_6_ monolayer possesses excellent stability by phonon spectrum and *ab initio* molecular dynamics (AIMD) calculations. Then, the electronic feature is investigated by band structure and carrier mobility. The optical performance of the C_2_B_6_ monolayer is addressed by light absorption spectrum and photocurrent testing.

## Computing method

All first-principle simulations were performed using the Vienna *ab initio* simulation package (VASP) (Oganov and Glass, 2006) using the DFT (Grimme et al., 2010; Van de Walle and Martin, 1989; Grest et al., 1981). The projector augmented wave potentials (PAW) were used in the calculations to demonstrate the core electrons (Kresse and Furthmüller, 1996a; Kresse and Furthmüller, 1996b; Blöchl, 1994). The Perdew–Burke–Ernzerhof (PBE) functional was conducted by the generalized gradient approximation (GGA) method (Kresse and Joubert, 1999; Perdew et al., 1996). The Heyd–Scuseria–Ernzerhof hybrid functional was explored to calculate a more accurate band structure and light absorption spectrum (Heyd et al., 2005; Heyd et al., 2003). The spin effect is not explored in the calculations because it has almost no effect on the electronic properties of the studied system, which is proved by the band structure demonstrated in Supplementary Figure S1 in Supporting Information. The energy cut-off was 550 eV. The Monkhorst–Pack *k*-point grid was set as 17 × 17 × 1 in the first Brillouin zone. The density functional perturbation theory (DFPT) was considered to obtain the phonon spectra by the PHONOPY code (Togo and Tanaka, 2015; Togo et al., 2008). Furthermore, the convergence for force was set as 0.01 eV Å^−1^, while the energy of the calculated system is set as 0.01 meV. The photocurrent of the C_2_B_6_ monolayer is calculated by Nanodcal software based on non-equilibrium Green’s function (NEGF) theory.

## Results and discussion

**FIGURE 1:**
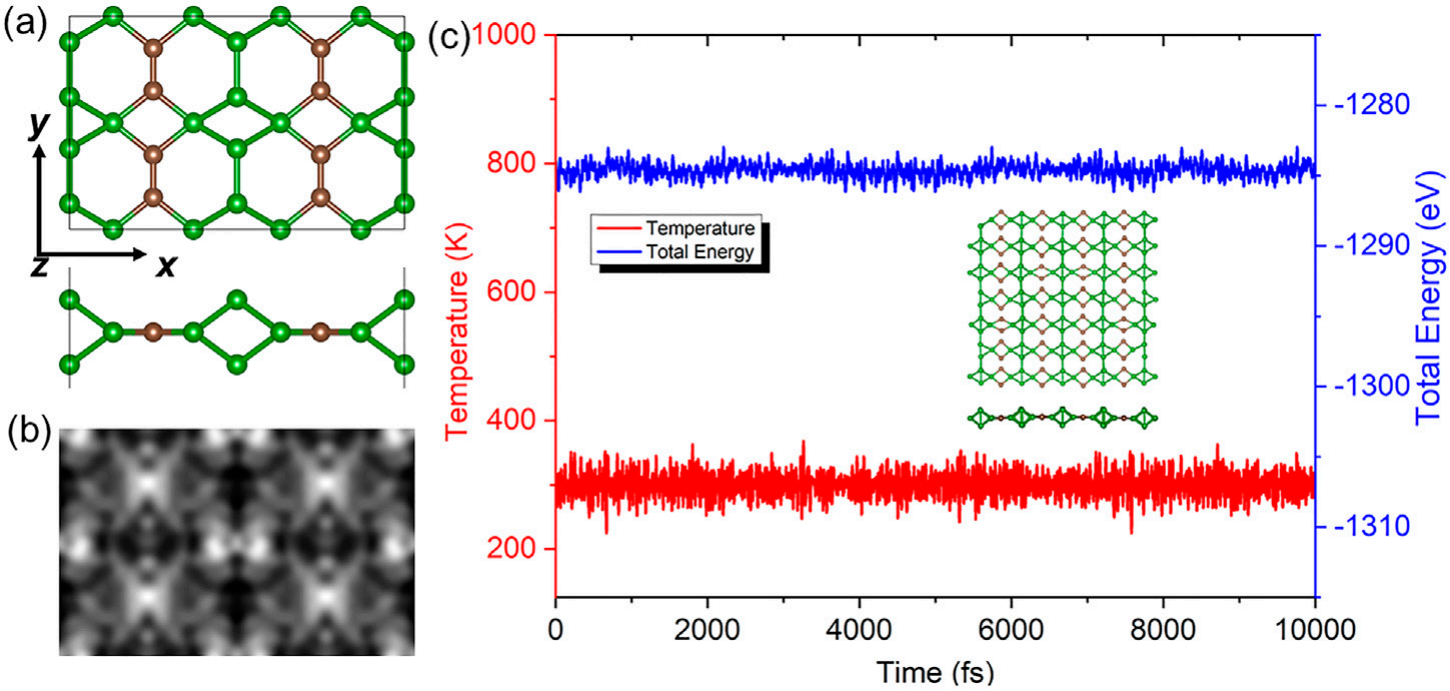
**(A)** Atomic structure and the **(B)** simulated STM configuration of the C_2_B_6_ monolayer at a voltage of −2 V. **(C)** Energy and the temperature of the C_2_B_6_ monolayer in the AIMD calculations. The inset is the relaxed structure of the C_2_B_6_ monolayer at 300 K for 10 ps. The green and the brown balls are B and C atoms, respectively.

First, the crystal structure of the C_2_B_6_ monolayer is predicted as a puckered unit-cell with the space group of *Pca*_*21*_, using the elemental mutation method from the prototype of the Li_*x*_B_*y*_ structure (Ren et al., 2022c), shown in Figure 1A. The optimized lattice parameters of the *x* and *y* in unit-cell of the C_2_B_6_ monolayer are 5.218 Å and 3.310 Å, respectively, which is comparable with the CS monolayer (Lv et al., 2020). The C–B bond and the C–C bonds are obtained as 1.59 Å and 1.32 Å, respectively.

The simulated STM configuration of the C_2_B_6_ monolayer is demonstrated in Figure 1B, which can provide a reference for experimental observations. The cohesive energy of the C_2_B_6_ monolayer is calculated as 6.516 eV/atom, which is obtained by (2*E*_C_ + 6*E*_B_ – *E*_CB_)/8, where *E*_C_, *E*_B_, and *E*_CB_ are the total energies of a C atom, a B atom, and the C_2_B_6_ monolayer, respectively. The calculated cohesive energy of the C_2_B_6_ monolayer is comparable with the predicted Li_*x*_B_*y*_ system (approximately 4.11–5.53 eV/atom) (Ren et al., 2022c) and the CB monolayer (approximately 6.13 eV/atom) (Ren et al., 2023a). It is also larger than that of the V–VI system (approximately 3.37–3.81 eV/atom) (Ren et al., 2022a), suggesting the stability of the C_2_B_6_ monolayer. The thermal stability of the C_2_B_6_ monolayer is estimated by the AIMD calculations using the Nosé−Hoover heat bath scheme (Nosé, 1984). The supercell of the C_2_B_6_ monolayer is constructed on a 7 × 4 × 1 grid to ensure the lattice translational constraints contain 192 atoms (Ren et al., 2020a). The C_2_B_6_ monolayer is relaxed at 300 K within 10 ps. After the completed simulations, the atomic structure of the C_2_B_6_ monolayer is still unscathed, as shown in the insets of Figure 1C. The temperature and energy of the AIMD for the C_2_B_6_ monolayer are also convergent, as shown in Figure 1C, which further provides evidence of stability. The C_2_B_6_ monolayer is also stable under 600 K, while the structure can be melted down at the temperature of 1,000 K, as demonstrated in the Supplementary Figures S2A, B, respectively.

**FIGURE 2:**
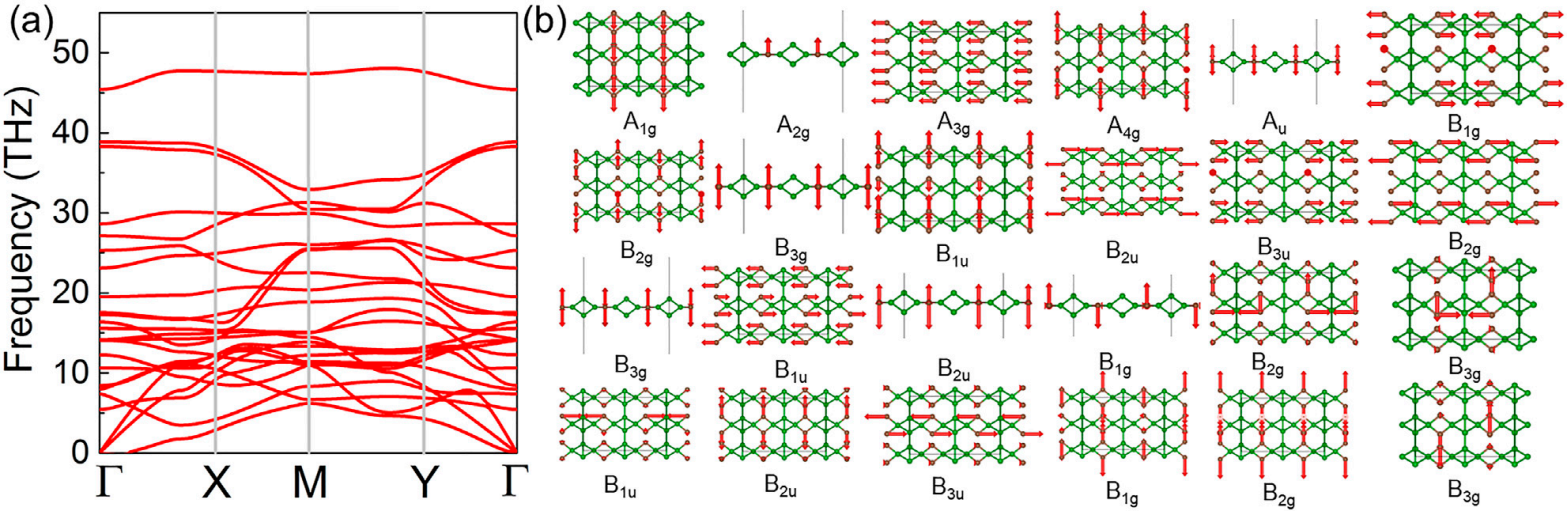
Calculated **(A)** phonon spectrum and the **(B)** atomic vibration mode of the C_2_B_6_ monolayer.

The dynamic stability of the C_2_B_6_ monolayer is investigated by phonon spectra, calculated in Figure 2A. One can see that there is no imaginary frequency in the phonon spectra of the C_2_B_6_ monolayer, implying the dynamic stability of the C_2_B_6_ system. The highest frequency of the optical branch can reach 45 THz, as shown in Figure 2A. Such maximal optical branch frequency is also comparable with the prototype (Li_*x*_B_*y*_ system), suggesting applications as efficient thermoelectric functional devices that can be tuned by the phononic crystal structure (Ren et al., 2020b). There are 24 degeneracy points at the Γ point. The lattice vibration mode of the C_2_B_6_ system at the Γ point for these 24 degeneracy configurations is studied, as shown in Figure 2B. All these optical phonons at the Γ point can be demonstrated as Equation 1:

$$\Gamma_{\text{optic}}=4A_{g}\;\left(R\right)+A_{u}\;\left(R\right)+3B_{1g}\;\left(\text{IR}\right)+3B_{1u}\;\left(R\right)+2B_{2g}\;\left(R\right)+3B_{2u}\;\left(\text{IR}\right)+B_{3g}\;\left(\text{IR}\right)+2B_{3u}\;\left(R\right),$$ (1)

where R, IR, and IN represent the optical phonon mode with Raman active, infrared active, and inactive, respectively. Interestingly, A_2g_, A_u_, B_3g_, A_2u_, and B_1g_ are out-of-plane vibration modes, and the others are a combined form.

**FIGURE 3:**
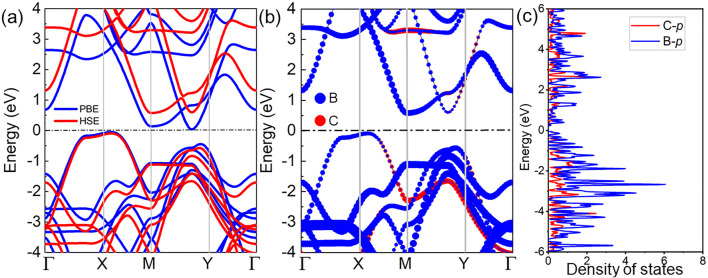
Obtained **(A)** band structure, **(B)** HSE06 calculated projected band structure, and **(C)** density of states of the C_2_B_6_ monolayer.

Then, the band structure of the C_2_B_6_ monolayer is investigated, as shown in Figure 3, by PBE and HSE06 methods. The C_2_B_6_ monolayer is a semiconductor with an indirect bandgap with the conduction band minimum (CBM) located between the M and Y points, while the valence band maximum (VBM) is set between the X and M points, demonstrated in Figure 3A. More interestingly, even though the wider bandgap is obtained by the HSE06 functional, it still presents as small as 0.671 eV, smaller than the As_2_X_3_ system (Zhao et al., 2023). It is worth noting that the ultra-narrow bandgap is also reported in the PbN/CdO heterostructure (approximately 0.128 eV). Such an ultra-narrow bandgap in the C_2_B_6_ monolayer facilitates rapid charge transitions and can serve as a potential efficient nanoelectronic device, optical device, and catalyst (Wang et al., 2017; Huang et al., 2024). The projected band structure of the C_2_B_6_ monolayer is calculated by the HSE06 in Figure 3B. The B atoms make an obvious and significant contribution to the energy band compared with the C atoms. The density of states (DOS) of the C_2_B_6_ monolayer is calculated in Figure 3C, which further proves that most of the energy level of the C_2_B_6_ system contributions come from B atoms.

**FIGURE 4:**
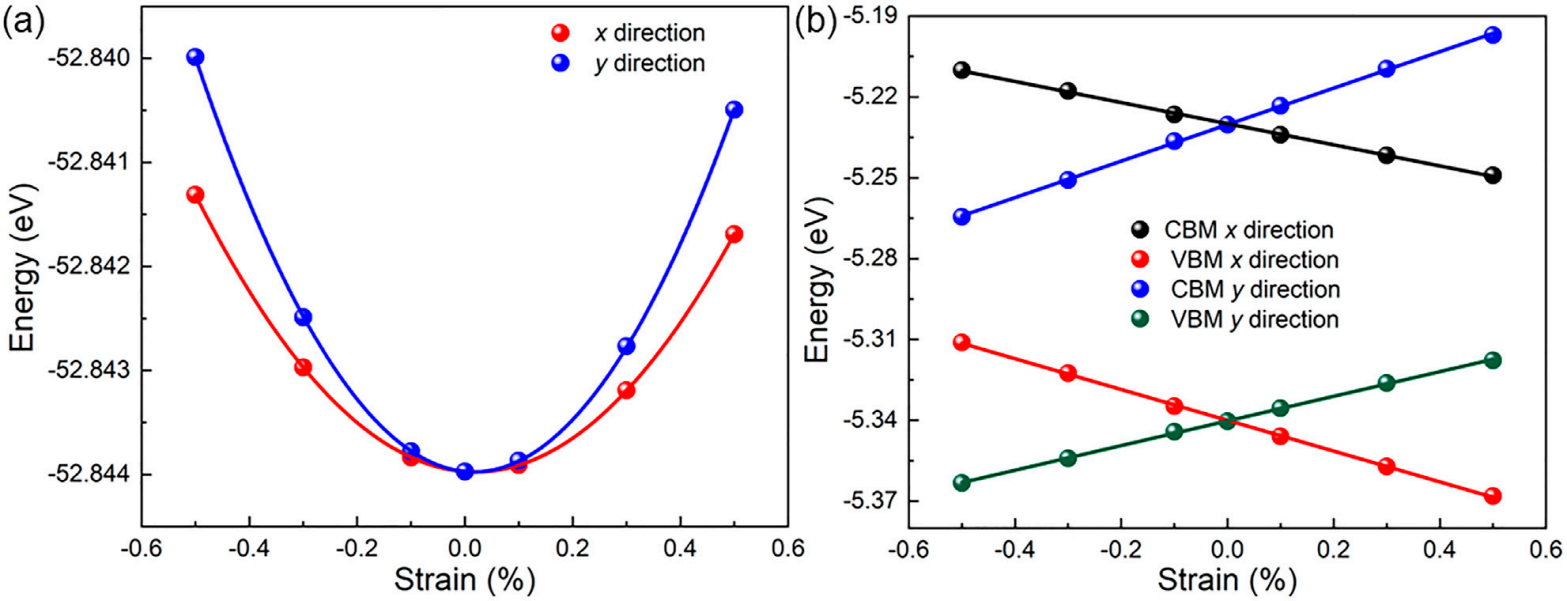
Difference of the **(A)** energy and the **(B)** band edge positions of the C_2_B_6_ monolayer in the *x* and *y* directions.

The carrier mobility is further explored, considering the ultra-narrow bandgap of the C_2_B_6_ monolayer for promising applications in nanodevices. The carrier mobilities of the electrons and holes in transport directions (*x* and *y* demonstrated in Figure 1A) are calculated by the Bardeen–Shockley theory demonstrated as Equation 2 (Van de Walle and Martin, 1989):

$$\mu =e\hbar^{3}C/\left(k_{B}Tm^{*}\sqrt{m_{x}^{*}m_{y}^{*}}D^{2}\right),$$ (2)

where *e* is the elementary charge, *ℏ* represents Planck’s constant, and *k*_B_ is the Boltzmann constant. The effective mass of the carriers, electrons, and holes is explained by m*, and the effective mass is obtained using Equation 3:

$$m^{*}=\hbar^{2}{\left(\frac{d^{2}E_{k}}{\;dk^{2}}\right)}^{-1},$$ (3)

where the wave vector is represented by the *k*. Electronic energy is demonstrated by the *E*_*k*_. *C* is the elastic modulus of the C_2_B_6_ monolayer calculated by *C* = [∂^2^ *E/*∂((*l*–*l*_*0*_ *)/l*_*0*_)^2^]/*S*_0_, where the free energy is *E*, and the original lattice constant and the difference by the strain are *l* and *l*_*0*_, respectively. *S*_0_ is the area of the C_2_B_6_ monolayer. The energy of the C_2_B_6_ monolayer under applied uniaxial is demonstrated in Figure 4A. One can see that the sensitivity of energy of the C_2_B_6_ monolayer to external strain in the *y* direction is significantly higher than that in the *x* direction, suggesting the higher elastic modulus of the *y* direction. Furthermore, *D* is used to show the potential constant of the C_2_B_6_ monolayer, which is calculated by *D* = Δ*E*_edge_/((*l*–*l*_*0*_ *)*/*l*_0_), where the Δ*E*_edge_ is the difference of the band edge by uniaxial strain along the transport directions. As shown in Figure 4B, when the strain is applied in the *y* direction, the CBM and the VBM can be increased linearly. Meanwhile, the CBM and the VBM of the C_2_B_6_ monolayer can be decreased linearly by the strain along the *x* direction, suggesting the external strain is an effective measure to tune the electronic properties of the C_2_B_6_ monolayer.

The calculated effective mass and deformation potential constant elastic modulus are demonstrated in Table. 1. It is worth noting that the effective mass of the C_2_B_6_ monolayer along the *y* direction is as low as 0.406 m*, suggesting higher carrier mobility. The sensitivity of edge energy to strain along *x* and *y* directions is similar for electrons and holes. The apparent mechanical anisotropy obtained from the elastic modulus of the C_2_B_6_ monolayer is calculated as 183 N⋅m^−1^ and 377 N⋅m^−1^, respectively, in the *x* and *y* directions. Thus, the pronounced anisotropic carrier mobility of the C_2_B_6_ monolayer is also obtained such that electrons 360 cm^2^⋅V^−1^⋅s^−1^ and 205 cm^2^⋅V^−1^⋅s^−1^ mobility in the *x* and *y* directions, respectively. More importantly, the C_2_B_6_ monolayer possesses ultrahigh hole mobility in the *y* direction of approximately 6,342 cm^2^⋅V^−1^⋅s^−1^. The difference of the carrier between the electron and hole in the *y* direction is also approximately 30 times, suggesting excellent promotion to separate the excited carriers. In addition, the electrons and holes show a favorable transport along the *x* and *y* directions, respectively. The obtained carrier mobility of the C_2_B_6_ monolayer is even higher than other popular 2D materials, such as the GaPS_2_Se_2_ monolayer (530 cm^2^⋅V^−1^⋅s^−1^) (Zhang Y. et al., 2022), the B_2_P_6_ monolayer (5,888 cm^2^⋅V^−1^⋅s^−1^) (Ren et al., 2021c), and MoSi_2_N_4_ (2,169 cm^2^⋅V^−1^⋅s^−1^) (Ren et al., 2023b) and is comparable with a Li_2_B_6_ monolayer (6,800 cm^2^⋅V^−1^⋅s^−1^) (Kai et al., 2018).

**TABLE 1:** Calculated effective mass (m*), deformation potential constant (*D*, eV), elastic modulus (C, N·m^−1^), and carrier (electron and hole) mobility (*μ*, cm^2^·V^−1^⋅s^−1^) of the C_2_B_6_ monolayer along the *x* and *y* directions.

Material (B)	Direction	Carrier	m*	*D*	*C*	*μ*
C_2_B_6_	*x*	Electron	1.221	−1.919	183	360
		Hole	1.743	−2.714		241
	*y*	Electron	2.121	2.771	377	205
		Hole	0.406	1.574		6,342

The light absorption performance of the C_2_B_6_ monolayer is further investigated by the absorption coefficient (*α*), which is calculated by Equation 4 (Zhang L. et al., 2022).

$$\alpha \left(\omega \right)=\frac{\sqrt{2}\omega }{c}{\left\{{\left[\varepsilon_{1}^{2}\left(\omega \right)+\varepsilon_{2}^{2}\left(\omega \right)\right]}^{1/2}-\varepsilon_{1}\left(\omega \right)\right\}}^{1/2},$$ (4)

where the *ε*_1_(*ω*) is the real part of the dielectric constant, and the *ε*_2_(*ω*) is the imaginary part. *ω* represents the angular frequency, and *c* is the speed of light in a vacuum. It is worth noting that *ε*_2_(*ω*) can be calculated by Equation 5 (Zhang et al., 2008):

$$\varepsilon_{2}\left(q\to O_{\hat{u}},\hbar \omega \right)=\frac{2e^{2}\pi }{\Omega \varepsilon_{0}}\sum_{k,v,c}|\left.\left\langle \Psi_{k}^{c}\right.\right|{\left.\hat{u}\cdot r\left|\Psi_{k}^{v}\right.\rangle \right|}^{2}\times \delta \left(E_{k}^{c}-E_{k}^{v}-E\right),$$ (5)

where $\Psi_{k}$ , $E_{k}$ , and $\hat{u}$ are used to explain the wave function, energy, and unit vector of the electric field of the incident light, respectively. Then, the superscripts (*v* and *c*) in the $\Psi_{k}$ and $E_{k}$ demonstrate the conduction and valence bands, respectively. Furthermore, *ε*(*ω*) = *ε*_1_(*ω*) + *iε*_2_(*ω*) can be used to calculate the complex dielectric function, and the Kramers–Kronig relation can define the real parts *ε*_1_ and *ε*_2_.

**FIGURE 5:**
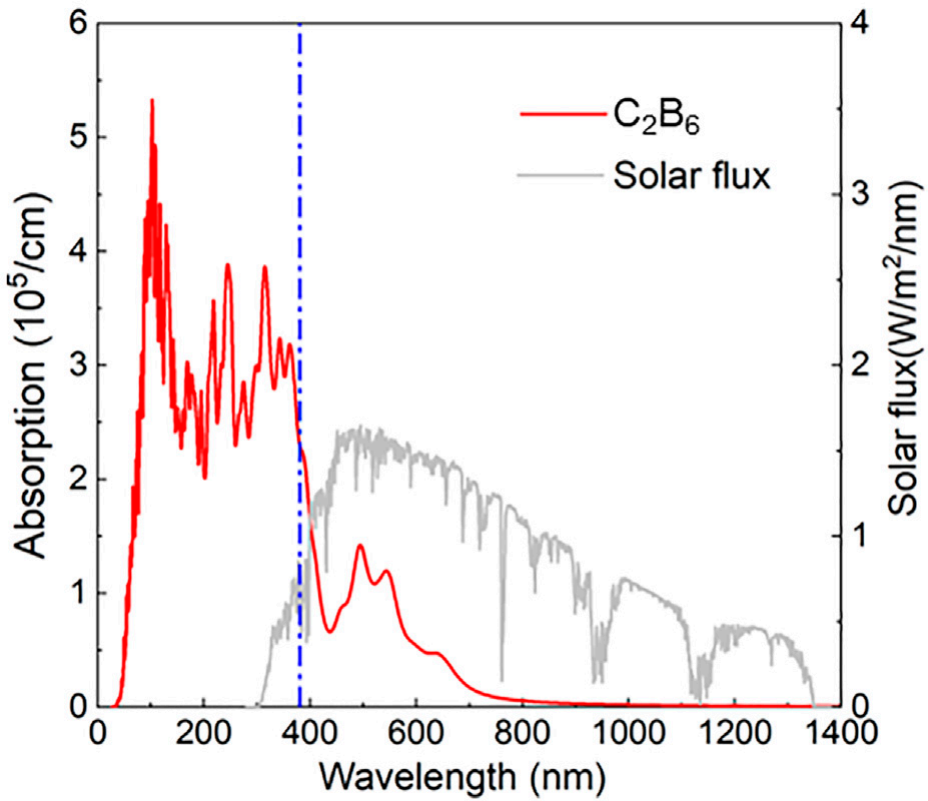
Obtained optical absorption spectrum of the C_2_B_6_ monolayer by the HSE06 method.

The obtained light absorption spectrum of the C_2_B_6_ monolayer is explained in Figure 5. The C_2_B_6_ monolayer shows excellent optical properties in the visible and near-ultraviolet regions with an absorption peak of approximately 3.566 × 10^5^ cm^−1^ and a wavelength of approximately 106 nm. The novel absorption coefficient at the visible light range is also obtained at approximately 9.578 × 10^4^ cm^−1^ with a wavelength of approximately 450 nm. Such an optical absorption peak of the C_2_B_6_ monolayer is also higher than other reported 2D materials, such as a CdO/arsenene heterostructure (8.47 × 10^4^ cm^−1^) (Ren et al., 2021d), GaN (4.00 × 10^4^ cm^−1^) (Ren et al., 2020c), and Mg(OH)_2_ (3.49 × 10^4^ cm^−1^) (Ren et al., 2019).

**FIGURE 6:**
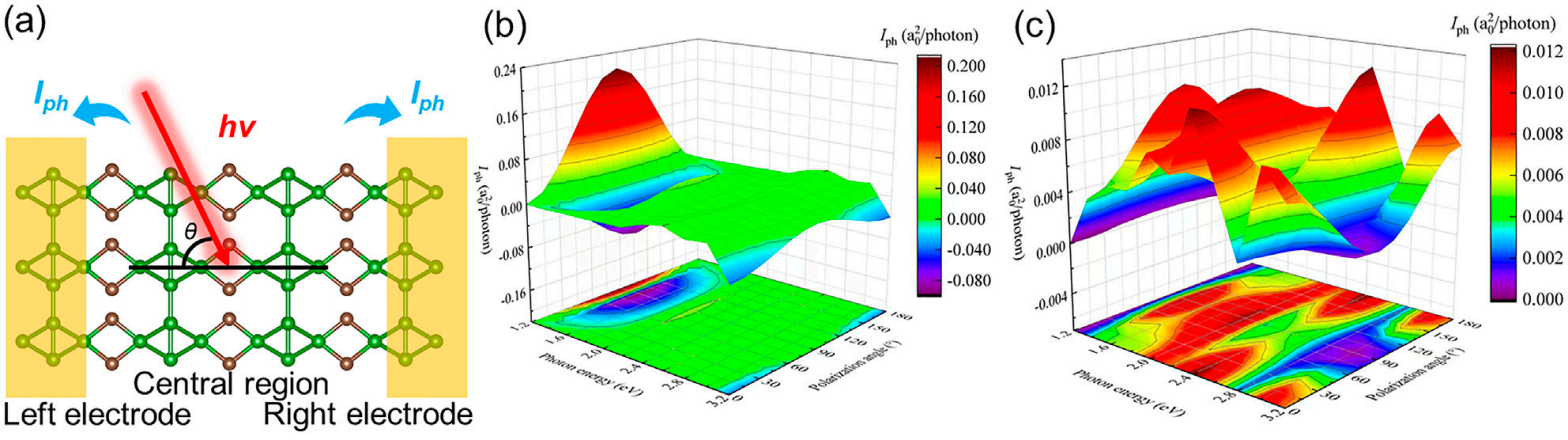
**(A)** Photodetector models and the calculated photocurrent along the **(B)** *x* and **(C)** *y* directions of the C_2_B_6_ monolayer.

The excellent carrier mobility and the optical performance of the C_2_B_6_ monolayer suggest potential applications as a photocurrent device. The model of the C_2_B_6_ monolayer used as a photocurrent nanodevice is illustrated in Figure 6A with two electrodes. The C and B atoms at the central region are excited by the linearly polarized light in the *z* direction and can induce the photon-generated carriers with the photocurrent flowing to the electrodes as *I*_ph_, which can be obtained as Equation 6 (Qin et al., 2024; Li et al., 2024):

$$J_{\text{ph}}=\frac{ie}{h}\int \text{Tr }\left\{\Gamma \left.\left[G^{<\left(\text{ph}\right)}+f\left(E\right)(G^{>\left(\text{ph}\right)}-G^{<\left(\text{ph}\right)}\right]\right\}dE,\right.$$ (6)

where $\Gamma =i\left(\Sigma_{R}-\Sigma_{L}\right)$ is the coupling of the center area and electrodes in the C_2_B_6_ monolayer. $\Sigma_{L}$ is the interactive self-energy of the left electrode, and the $\Sigma_{R}$ is interactive self-energy of the right one. *f*(*E*) is the Fermi−Dirac distribution. Green’s functions for photon−electron interactions are presented by $G^{>\left(\text{ph}\right)}$ . *J*_ph_ is normalized by *I*_ph_ = *J*_ph_/e*I*_ω_, and the *I*_ω_ demonstrates the photon flux. The calculated unit for the photocurrent is a0 2/photon, where the a^0^_2_ is used to explain the Boreal radius. The photocurrent is also dependent on the photon energy and polarization angle of the C_2_B_6_ monolayer; thus, the photocurrent C_2_B_6_ monolayer is calculated with a different angle and intensity of light incidence in Figure 6B. One can see that the maximal *I*_ph_ of the C_2_B_6_ monolayer is approximately 0.24 a^0^_2_/photon with the energy and the polarization angle of approximately 1.2 eV and 90°, respectively. Note that the anisotropy of carrier mobility also implies different photocurrents in the *x* and *y* directions. The obtained maximal *I*_ph_ of the C_2_B_6_ monolayer along the *y* direction is demonstrated in Figure 6C as approximately 0.012 a^0^_2_/photon at the polarization angle of 90° with the energy of approximately 2.4 eV. The obtained photocurrent of the C_2_B_6_ is comparable with the other reported 2D materials, for example, MoSSe (0.88 a^0^_2_/photon) (Cui et al., 2024), WSe_2_/MoSe_2_ (0.65 a^0^_2_/photon) (Sun et al., 2023) and MoS_2_/WSSe (0.71 a^0^_2_/photon) (Sun et al., 2023) linearly polarized lights.

Furthermore, the dependence of the photocurrent of the C_2_B_6_ on angle and energy is also different along the *x* and *y* directions compared with Figures 6B, C. The photocurrent of the C_2_B_6_ can be decreased with increasing energy, and the vertical illumination method can obtain the maximal photocurrent along the *x* direction. Differently, the optimum photocurrent of the C_2_B_6_ can be induced by the horizontal irradiation method. With increasing energy, there is no unified trend of change for the photocurrent of the C_2_B_6_ along the *y* direction. Thus, the photocurrent direction can be effectively controlled by adjusting the incident angle, another promising attribute for a photoelectric device. When the C_2_B_6_ monolayer is illuminated, the photogenerated electrons can move quickly to the conduction band due to the narrow bandgap, inducing the valence band with photogenerated holes. Under the drive of a photocurrent, photogenerated electrons and holes can be rapidly separated due to the strong anisotropy of the mobility, implying that C_2_B_6_ is a potential high-efficiency photocatalyst.

## Conclusion

In summary, a C_2_B_6_ monolayer is proposed with inherent stability. The puckered crystal structure of the C_2_B_6_ monolayer presents semiconductor properties with an ultranarrow indirect bandgap of approximately 0.671 eV, while the ultrahigh hole mobility is calculated as 6,342 cm^2^⋅V^−1^⋅s^−1^ in the suitable direction. The calculated anisotropic carrier mobility of the electrons and holes in the C_2_B_6_ monolayer demonstrates the advantages of carrier separation for use as a photocatalyst. Finally, the excellent light absorption and the photocurrent are also addressed, demonstrating the potential applications for photocatalytic, photovoltaic and optical and cold chain electronic devices.

## Data Availability

The original contributions presented in the study are included in the article/Supplementary Material; further inquiries can be directed to the corresponding authors.
