# Supplementary material for: Predicting the characteristics of a C2B6 monolayer with ultrahigh carrier mobility
Source: Front Chem. 2024 Oct 24;12:1482006. doi: 10.3389/fchem.2024.1482006 (PMC11540763; doi:10.3389/fchem.2024.1482006)
Supplement: Supplementary file 1 [file DataSheet1.docx]

**Supporting Information**

**Predicting the characteristics of a C_2_B_6_ monolayer with ultrahigh carrier mobility**

Ping Xu^1^, Zhengyang Zhu^2^, Ruxin Zheng^3^, Qingyun Sun^4,5^*, Zhen Ma^6^, Weihua Mu^7,^* and Zhen Cui^8,^*

^1^Jiangsu Vocational College of Agriculture and Forestry, Jiangsu, 212400, China

^2^School of Mechanical Engineering, Wanjiang University of Technology, Maanshan 243031, China

^3^School of Mechanical Engineering, Southeast University, Nanjing 211189, China

^4^School of Mechanical and Electronic Engineering, Nanjing Forestry University, Nanjing, Jiangsu 210037, China

^5^Nanjing Boya Intelligent Technology Co., Ltd, Jiangsu, 210042, China

^6^School of Agricultural Engineering, Jiangsu University, Zhenjiang, 212013, China

^7^Wenzhou Institute, University of Chinese Academy of Sciences, Wenzhou, China

^8^School of Automation and Information Engineering, Xi’an University of Technology, Xi’an 710048, China

The band structure of the C_2_B_6_ monolayer with the spin effect is calculated in the Figure S1a, one can see that the band energy contributed by spin-up and spin-down is the same. Furthermore, the band structure of the C_2_B_6_ monolayer with and without spin effect is also the same in the Figure S1b, therefore, the spin effect can be ignored in the calculations.


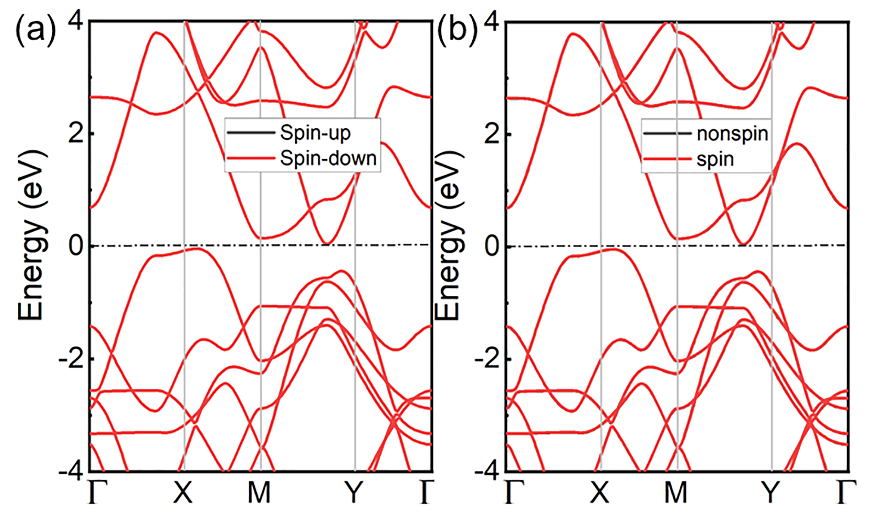


**Figure S1.** The band structure of the C_2_B_6_ monolayer obtained by (a) spin-up, spin-down and the (b) the comparation of the band structure calculated by nonspin and spin using PBE method.

The temperature and energy of the AIMD for the C_2_B_6_ monolayer at the 600 K and 1000 K are shown as Figure S2a and b, respectively. One can see that the C_2_B_6_ monolayer is still stable at 600 K.


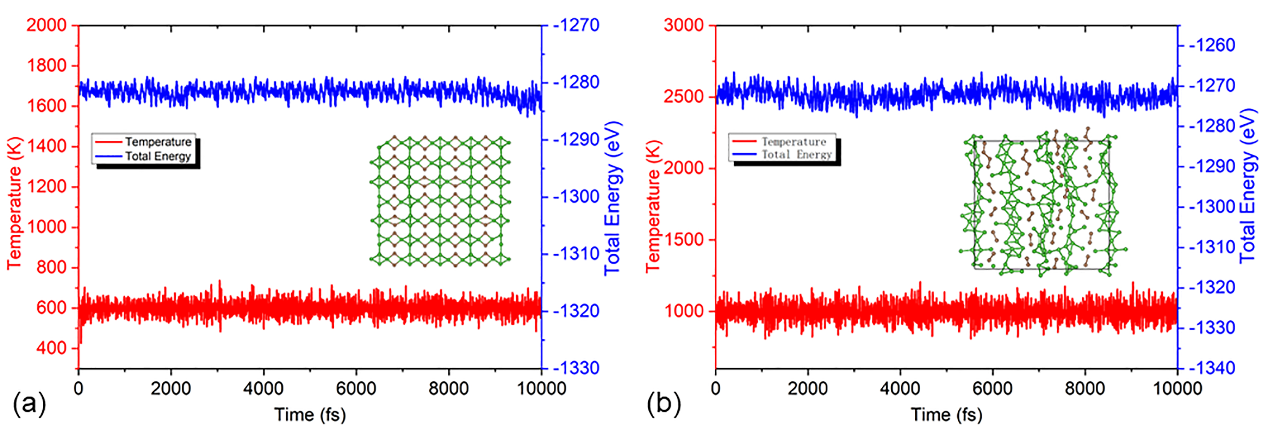


**Figure S2.** The energy and the temperature of the C_2_B_6_ monolayer in the AIMD calculations, the inset is the relaxed structure of the C_2_B_6_ monolayer at (a) 600 K and (b) 1000 K for 10 ps.
